# Definitive Radiotherapy for Locally Advanced Merkel Cell Carcinoma of the Head and Neck Region: A Case Report

**DOI:** 10.7759/cureus.6270

**Published:** 2019-12-01

**Authors:** Aron Gortman

**Affiliations:** 1 Radiation Oncology, Lismore Base Hospital, North Coast Cancer Institute, Lismore, AUS

**Keywords:** merkel cell carcinoma, radiation therapy, skin cancer, histopathology, head & neck, case report, diagnosis

## Abstract

Merkel cell carcinoma (MCC) is a highly aggressive neuroendocrine malignancy of skin origin. It is most commonly managed with upfront surgical resection which is then followed by radiotherapy as soon as possible postoperatively. Radiotherapy alone has been used in inoperable cases and in cases of patient preference to omit surgical management. MCC is a very radiosensitive disease, and an ideal location for definitive radiotherapy is the head and neck region (which represents the majority of cases), where resection often leads to reduced outcomes given the number of critical structures in the region and the inability of surgery to achieve the excision margins required for the aggressive nature of the disease without leading to severely impacted cosmesis and function. This case study looks at an elderly woman who was treated with definitive dose fractionation (to a total dose of 60 Gray in 30 fractions) radiotherapy to the head and neck region for Stage III, T1N2M0 disease, following rapid loco-regional relapse after surgical wide local excision. Two years postdefinitive radiotherapy, she has had complete loco-regional control both clinically and radiologically.

## Introduction

Merkel cell carcinoma (MCC) is a highly aggressive neuroendocrine malignancy of skin origin [1]. It is uncommon in most parts of the world but not uncommon in Australia, with an approximate incidence of 1.6 per 100,000 individuals in the state of Queensland, at least double that of the rest of the world [1]. MCC is more commonly seen in the elderly [1]. It is likely that the high UV index, coupled with a population with fairer skin and consistent sunlight exposure throughout life, are key contributors to the higher incidence of MCC seen in Australia [1]. While there is no firmly established general first-line treatment (e.g. based on the results of a randomized controlled trial), treatment tends most commonly to be upfront surgery, as patients are initially referred to a surgeon for biopsy/excisional biopsy, which is then followed as soon as possible by postoperative radiotherapy [2]. Definitive radiotherapy is also a very good option given the high radiosensitivity of MCC, and can be utilized based on anatomical location, specifically the head and neck region (the most common region of primaries, representing up to 60% of cases), where resection often leads to reduced outcomes given the number of critical structures and the inability of surgery to achieve the excision margins required for the aggressive nature of the disease without leading to severely impacted cosmesis and function [3-4]. Definitive radiotherapy may also be used in patients not deemed operable candidates [4]. Other scenarios include patient preference for radiotherapy only or refusal to undergo surgery [4]. Total doses of up to and higher than 60 Gray have been reported in the definitive setting [5]. This case study looks at an elderly woman who was treated with definitive dose fractionation radiotherapy to the head and neck region for Stage III, T1N2M0 disease, following loco-regional relapse after surgical wide local excision. Two years postdefinitive radiotherapy, the patient has had complete loco-regional control both clinically and radiologically.

## Case presentation

An 82-year-old Australian female was referred by her general practitioner to a general surgeon for biopsy +/- excision of a clinically up to 15 mm lesion of the mid-nose, which had been rapidly enlarging in size over the previous two to three weeks. The lesion was subsequently managed surgically by wide local excision and closed with an advancement flap. The histopathology report described an ulcerated tumor composed of undifferentiated small cells invading into the superficial subcutis. The cells stained strongly positive for cytokeratin-20 (CK-20), chromogranin, synaptophysin, and CAM 5.2; and stained negative for both thyroid transcription factor-1 (TTF-1) and CK-7. The immunohistochemical profile was therefore consistent with diagnosis of MCC [2]. In terms of the surgical margins, the peripheral margins were clear by at least 3 mm and the deep margin was seen to be clear by approximately 1.5 mm. There was neither lymphovascular space invasion nor perineural invasion seen.

The case was subsequently discussed at the hospital’s multidisciplinary (MDT) specialities meeting, with the consensus recommendation for discussion of postoperative radiotherapy under radiation oncology. Review of staging CT imaging three weeks postexcision showed features concerning for local recurrence just adjacent to the surgical scar site on the left side of the nasal bridge, or left nasal region of the maxilla. On review in the radiation oncology clinic, the suspicious region had a palpable nodule underneath it with firm and hard consistency, felt to be just less than 5 mm in diameter. Fluorodeoxyglucose-18 positron emission tomography (F-18 FDG PET) staging scan was subsequently organized in addition to a biopsy from this suspicious nodule.

The PET scan confirmed the clinical findings of loco-regional recurrence postsurgery, with a moderately glucose avid >10 mm (in short axis) subcutaneous nodule over the left anterior maxilla adjacent to the nose (as described clinically), an avid >10 mm (in short axis) enlarged left Level IB node just anterior to the left submandibular gland, and an equivocal <10 mm (in short axis) right Level IB node adjacent to the submandibular gland. 

Fine needle aspiration (FNA) of the left cheek lesion showed specimen demonstrating paranuclear dot positivity for CK-20 and positivity for synaptophysin and chromogranin, and CAM5.2; TTF-1 and CK7 immunohistochemistry were again negative, consistent with MCC [2].

**Figure 1:**
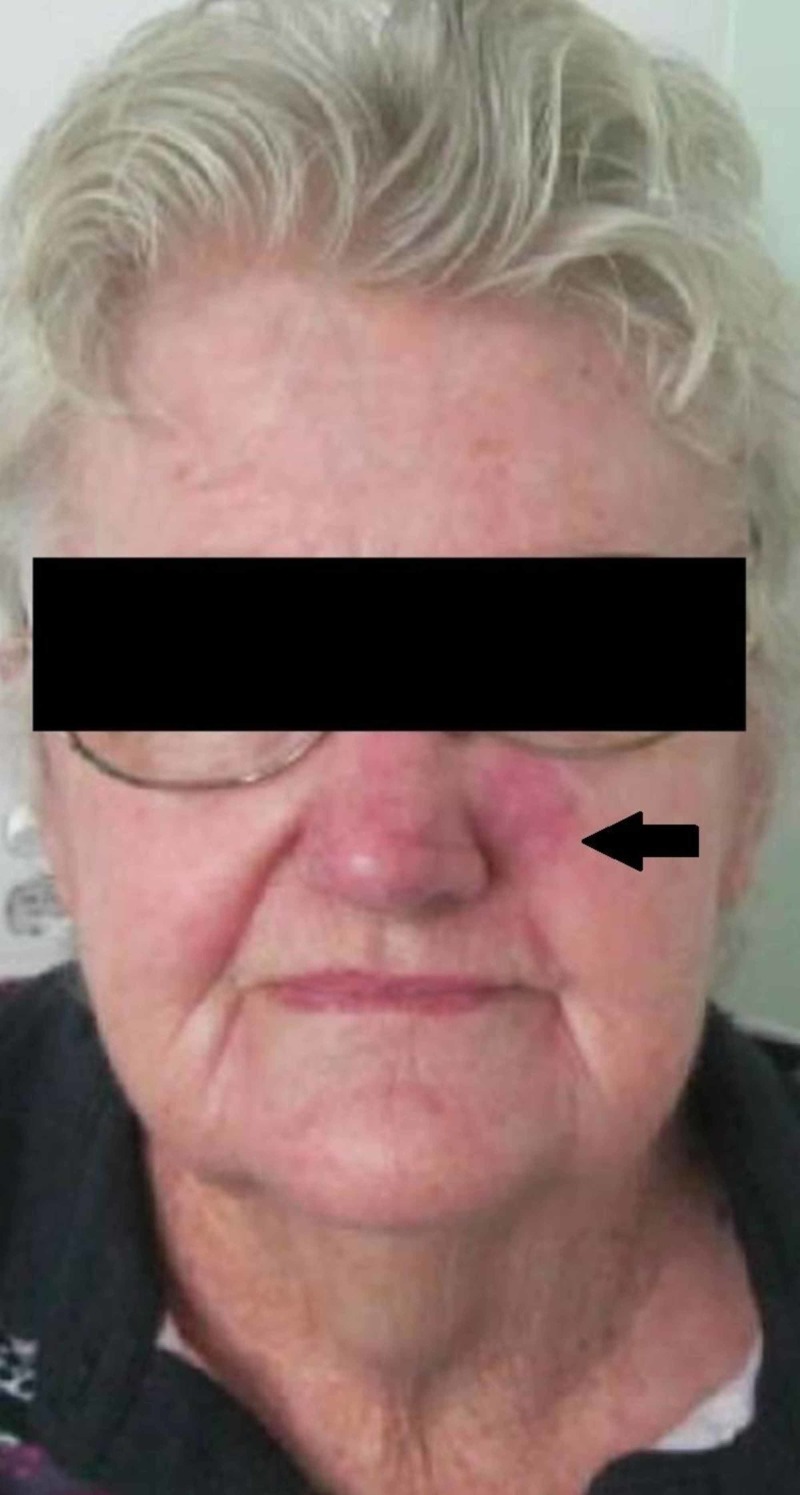
Pretreatment photograph in radiation oncology clinic showing lesion of skin of left nasal region of maxilla.

The case was then further discussed at a large metropolitan centre Head and Neck MDT. By that time, the recurrent nodule on the left nasal region of the maxilla had grown to up to 3 cm with overlying erythema, and on examination there was palpable Level II cervical nodal regions bilaterally (Figure 1). The MCC was therefore now consistent with T1N2M0, Stage III disease [6]. The consensus recommendation from the MDT panel was for definitive intent radiotherapy to the nose, to the bilateral neck, and to consider the bilateral cheeks. The patient’s ECOG status was 1.

**Figure 2:**
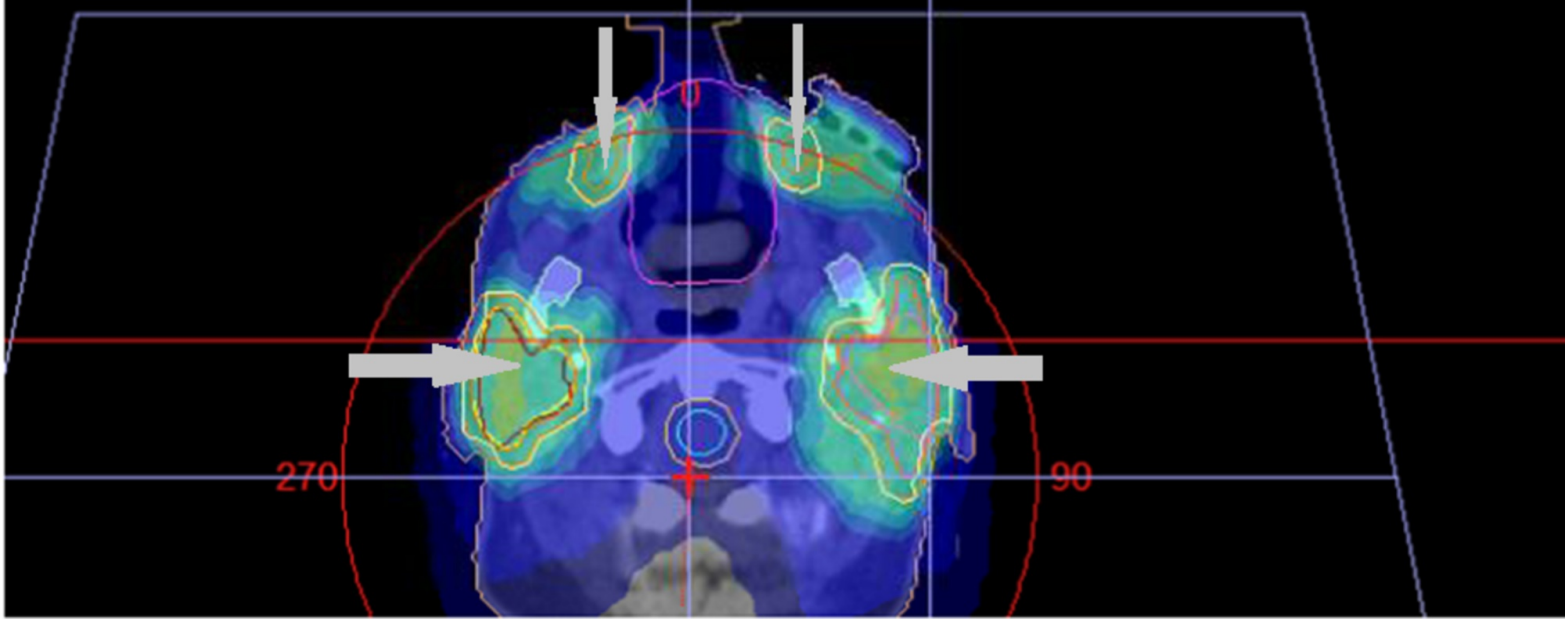
Axial view of the Level I cervical nodes (thicker arrows) and intervening lymphatics (thinner arrows). Various isodose (areas receiving equal doses of radiation) washes represented in dark blue (lower percentage of the total dose), lighter blue, and yellow (higher percentage of the total dose) within the target volumes indicated by the arrows. The spinal cord is also outlined in this figure.

**Figure 3:**
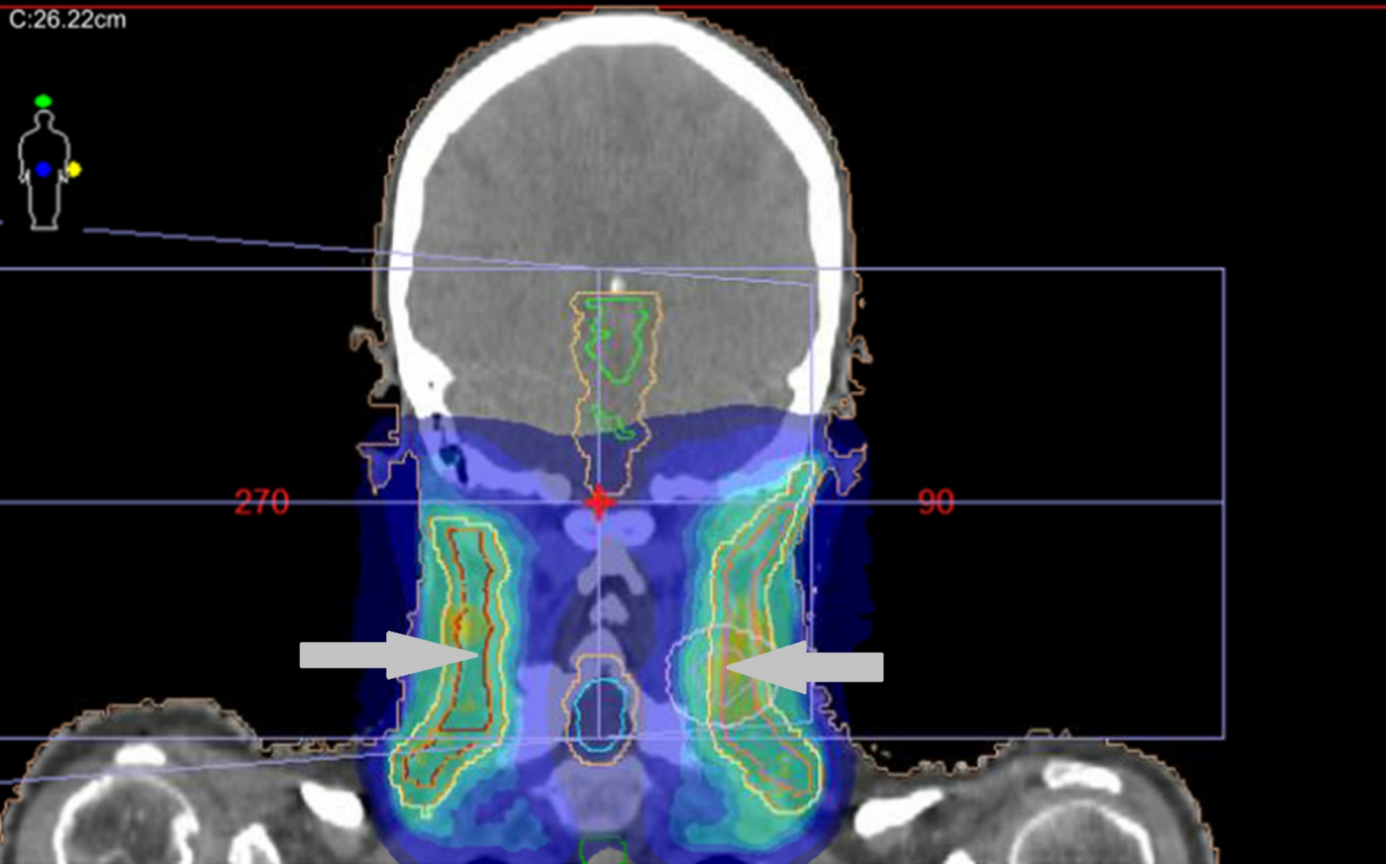
Coronal view illustrating the bilateral neck treatment volumes. Various isodose (areas receiving equal doses of radiation) washes represented in dark blue (lower percentage of the total dose), lighter blue, and yellow (higher percentage of the total dose) within the target volumes indicated by the arrows. The spinal cord and brainstem are also outlined in this figure.

**Figure 4:**
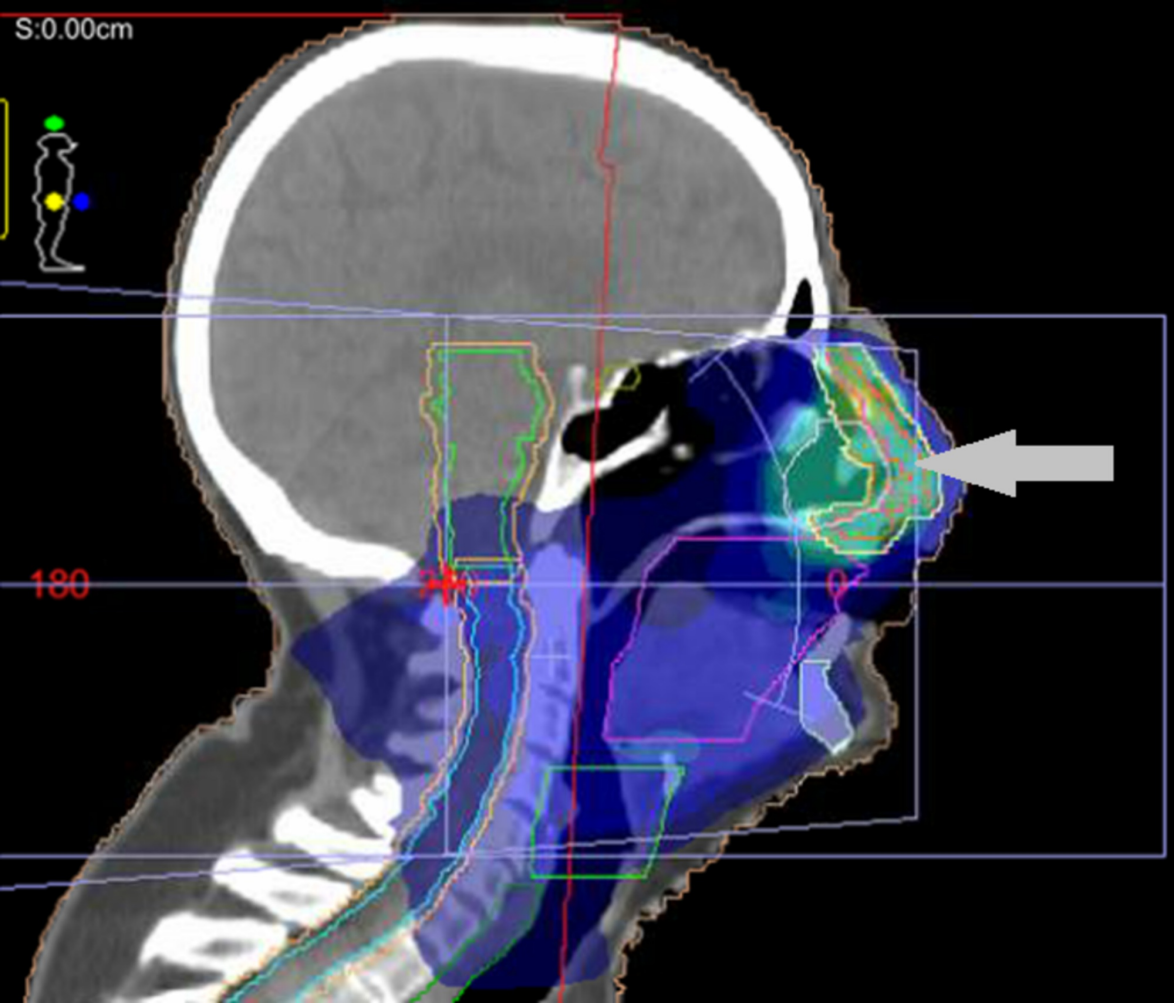
Sagittal view illustrating left nasal region of maxilla treatment volume. Various isodose (areas receiving equal doses of radiation) washes represented in dark blue (lower percentage of the total dose), lighter blue, and yellow (higher percentage of the total dose) within the target volumes indicated by the arrows. The brainstem and the oral cavity is also outlined in this figure.

The patient was treated to a total dose fractionation of 60 Gray in 30 fractions, 2 Gray per fraction, daily for six weeks, with 6 megavoltage (MV) photons, using volumetric modulated arc therapy (VMAT). A simultaneous integrated boost (SIB) technique was employed, in which the left nasal region of the maxilla and bilateral Level IB cervical nodes received 60 Gray in 30 fractions, and a total dose of 50 Gray was administered to the bilateral facial nodes, resected nasal disease (with a margin), bilateral parotids, and intervening lymphatics. A 1 cm custom tissue equivalent material (commonly referred to as bolus) was applied over the left nasal region. A mouthbite was used, and the nose was packed with gauze. A gross tumour volume (GTV) representing the recurrent nodule on the left nasal region of the maxilla was outlined; there were further expansions with a clinical tumour volume (CTV) expansion of up to approximately 5 mm and a planning target volume (PTV) expansion of up to 10 mm for the structures treated to 50 and 60 Gray as described above (Figures 2-4).

 

The patient was also referred for a discussion of concurrent carboplatin chemotherapy under medical oncology [2]. Carboplatin was poorly tolerated, leading to a hospital admission, and chemotherapy was subsequently withdrawn after a single cycle. At that point, there was already noted in the clinic to be a marked reduction in the size of the lesion with radiotherapy. By the completion of radiotherapy, there was essentially complete remission clinically of the lesions of both the left nasal region of the maxilla and the bilateral Level II cervical nodes.

Both parotids were included in the treatment field. The mean dose to the bilateral parotid glands was between 53 and 54 Gray. Approximately 23% of the total volume of the mandible received a dose of 50 Gray, with no ‘hot spots’ (as defined by ICRU50 as a volume outside the PTV receiving dose greater than 100% of the specified PTV dose) in the mandible. Acute toxicities [defined by Common Terminology Criteria for Adverse Events (CTCAE) Version 4 as occurring within 90 days of treatment] reported by the patient included Grade I xerostomia with reduced saliva without significant dietary alteration, Grade I dermatitis with mild erythema in the treatment field, Grade I fatigue, and Grade I facial oedema with localized edema. Late toxicities, defined by CTCAE as occurring more than three months after the completion of radiotherapy, included the persisting or consequential Grade I xerostomia; and Grade I facial edema with localized edema. The relatively mild reported xerostomia by the patient both in the acute and late stages was anomalous given that there was no sparing of either parotid gland from the target volumes. However, approximately 18 months postcompletion of radiotherapy, a lower right premolar tooth was extracted by the patient’s local dentist. The cause was thought by the treating dentist to be in the context of xerostomia and dose received to the mandible.

**Figure 5:**
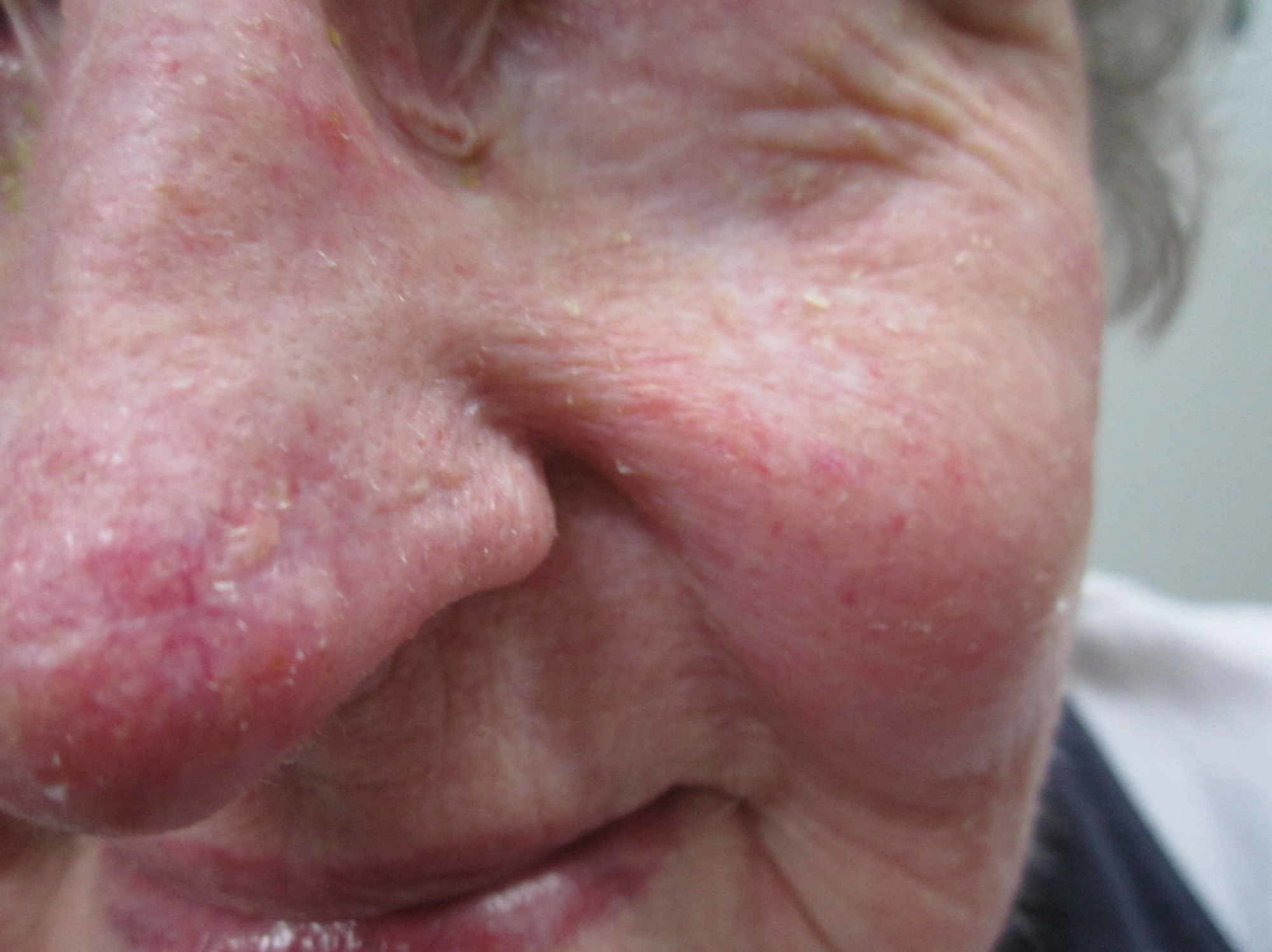
Clinical photo showing full resolution of lesion of skin left nasal region of maxilla.

The PET to assess treatment response three months postcompletion of radiotherapy demonstrated a complete metabolic response. All subsequent imaging have demonstrated complete response with no evidence of recurrence or distant disease. This has correlated with findings on clinical examination on three-six monthly reviews (Figure 5). The patient remains well and has 6-12 monthly interval CT re-staging.

## Discussion

In this case report, an elderly female with MCC of the head and neck region was treated to definitive dose radiotherapy following rapid loco-regional recurrence after initial management with surgery. The patient remains well, more than two years postcompletion of radiotherapy, and with no Grade II or higher late toxicities as per CTCAE criteria. This case report also highlights the importance of rapid treatment with radiotherapy of MCC following biopsy (or surgical specimen) proven diagnosis.

An Australian retrospective study reported on records of 43 patients treated with definitive radiotherapy, either at diagnosis or in the relapse setting, between 1993 and 2007; again, the head and neck region was the most commonly treated site [7]. Definitive radiotherapy, in a fractionation schedule of up to 55 Gray in 20-25 fractions used in the study, showed very good in-field control rates, of around 75% [7]. The majority of patients in that study presented with earlier stage disease than that seen in this case study.

The use of adjuvant or prophylactic nodal irradiation and the role of sentinel lymph node biopsy and mapping have been discussed and described in the literature in Stage I disease [8]. In this Stage III case, the clinically involved cervical nodal regions and the draining lymphatics were treated.

There is an established relationship between MCC and the Merkel cell polyomavirus (MCPyV), thought to be an agent in MCC development, and which may be further potentiated in immunocompromise, including organ transplantation, HIV infection/AIDS, autoimmune diseases, and lymphoproliferative disorders, particularly B cell lymphoma [9]. Immunocompromised individuals are strongly considered to have a significantly elevated risk of developing MCC, as well as earlier and more aggressive disease, according to reports that quantify risk [9]. Eliciting a history of immuncompromise and its treatment course, and pharmacological management of this including full history of immunosuppressant and immunomodulating agent use is therefore critical in the clinic, and these patients should be seen as soon as possible in the MDT setting following diagnosis [9]. The programmed death-ligand 1 (PDL-1) inhibitor Avelumab has shown some promise in the metastatic setting as a potential first-line immunotherapy agent [10].

This case should hopefully provide further evidence that in some or perhaps most cases of MCC, and particularly in the head and neck region, (a) radiotherapy alone can provide definitive management, based on patient, tumour, and treatment characteristics, (b) and that radiotherapy can be delivered with tolerable side effects even in an extended treatment field.

It is conceivable that in the not distant future, following discussion at an MDT, a patient could potentially be referred directly to both a radiation oncologist and to a surgeon following diagnosis of a biopsy-proven MCC, to discuss the options of either definitive radiotherapy or of upfront surgery followed by postoperative radiotherapy, should a patient wish to pursue active management.

## Conclusions

This report presents a case of an elderly woman who was treated with definitive dose fractionation (to a total dose of 60 Gray in 30 fractions) radiotherapy to the head and neck region for Stage III, T1N2M0 disease, following rapid loco-regional relapse after surgical wide local excision. Two years postdefinitive radiotherapy, she has had complete loco-regional control both clinically and radiologically. This case will hopefully add to a body of evidence that MCC of the head and neck region managed with radiotherapy alone can provide strong loco-regional control and that radiotherapy can be delivered with tolerable side effects in an extended treatment field in this setting. In addition, the case has highlighted the known radiosensitivity of MCC as well as necessity for rapid referral to radiation oncology following a diagnosis of MCC.
